# Effects on the Mechanical Properties of Nacre-Like Bio-Hybrid Membranes with Inter-Penetrating Petal Structure Based on Magadiite

**DOI:** 10.3390/ma12010173

**Published:** 2019-01-07

**Authors:** Mingliang Ge, Xubin Wang, Mingyi Du, Guodong Liang, Guoqing Hu, Jahangir Alam S.M.

**Affiliations:** 1 Key Laboratory of Polymer Processing Engineering of Ministry of Education, National Engineering Research Center of Novel Equipment for Polymer Processing, School of Mechanical & Automotive Engineering, South China University of Technology, Guangzhou 510640, China; gml@scut.edu.cn (M.G.); xubinwang66@163.com (X.W.); dumingyisir@163.com (M.D.); gqhu@scut.edu.cn (G.H.); 2 PCFM and GDHPPC Labs, School of Materials Science and Engineering, Sun Yat-Sen University, Guangzhou 510275, China; lgdong@mail.sysu.edu.cn; 3 Department of Robotics & Mechatronics Engineering, University of Dhaka, Dhaka 1000, Bangladesh; 4 Department of Computer Science & Engineering, Jessore University of Science and Technology, Jessore 7408, Bangladesh

**Keywords:** magadiite, chitosan, nacre-like, inter-penetrating petals, bio-hybrid membranes

## Abstract

Rigid biological systems are increasingly becoming a source of inspiration for the fabrication of the advanced functional materials due to their diverse hierarchical structures and remarkable engineering properties. As a bionic biomaterial with a clear layered structure, excellent mechanical properties, and interesting rainbow colors, nacre has become one of the most attractive models for novel artificial materials design. In this research paper, the tough and strong nacre-like bio-hybrid membranes with an interpenetrating petals structure were fabricated from chitosan (CS) and magadiite (MAG) clay nanosheets through the gel-casting self-assembling method. The analyses from X-ray diffraction (XRD), scanning electron microscope (SEM), and observations of water droplets on membranes indicated that the nacre-like hybrid membranes had a layered compact structure. Fourier transforms infrared spectroscopy (FTIR) analyses suggested that the CS molecular chains formed chemical bonds and hydrogen bonds with MAG layers. The inter-penetrating petal layered structure had a good effect on the mechanical properties of a nacre-like bio-hybrid membranes and the tensile strength of the hybrid membranes could reach at 78.6 MPa. However, the transmission analyses of the results showed that the hybrid membranes still had a certain visible light transmittance. Finally, the hybrid membranes possessed an intriguing efficient fire-shielding property during exposure to the flame of alcohol burner. Consequently, the great biocompatibility and excellent mechanical properties of the bio-hybrid membranes with the special interpenetrating petals structure provides a great opportunity for these composites to be widely applied in biomaterial research.

## 1. Introduction

Scientists often get inspiration from natural biology to make new materials. Natural nacre, consisting of 95 wt % aragonite and 5 wt % biological macromolecules, possesses remarkable mechanical performance [1]. Although nacre mostly consists of a fragile mineral, its toughness is three orders of magnitude higher than that of calcium carbonate in the form of aragonite [2]. The tensile strength and Young’s modulus of nacre are in the range of 80–135 MPa and 60–70 GPa, respectively [3,4]. Scientists discovered through research that the proteinaceous layers play a fundamental role in the formation of the aragonite tablets, which is a multi-layer complex arrangement of the biopolymer. The crystalline aragonite tablets were the rigid building blocks of the structure and present all the same crystallographic orientation because of the inter-tile connections through mineral bridges across the porous polymeric layers [5]. Such natural materials were characterized by ordered artworks and large fractions of hard, reinforcing segments with a minor amount of soft, energy-absorbing, lubricating biopolymers. The interface between hard and soft was well-controlled [6,7]. In short, the scientists describe this particular structure of nacre as a “bricks-and-mortars” nanostructure between the alternating layers of protein and aragonite tablets, which is why nacre has outstanding strength, toughness, stiffness, and impact resistance [8].

Due to its extraordinary mechanical properties, nacre has inspired the fabrication of bio-inspired, layered nanocomposite materials [9]. In the past few decades, works of literature had synthesized a lot of nacre-like materials with excellent mechanical properties, which was depend on material types and manufacturing process [10]. Sheikh et al. [11] reported calcium carbonate and electrostatically stabilized Nano crystalline cellulose (ENCC) as well as dicarboxylated cellulose (DCC) resulted in macroscale nacre-like sheets of vaterite using a bioinspired mineralization method to successfully address key biomimetic material design concerns. Zhang et al. [12] successfully fabricated a novel composite membrane Nafion-[PDDA/ZrP](n) with nacre-like nanostructures using an layer-by-layer (LBL) method, which presented excellent proton exchange performance for vanadium redox flow battery applications. Sarin et al. [13] applied the evaporation-induced self-assembly method to produce a layered crystalline polymorph film in the presence of chitosan and clay nanoplatelets. Zhao et al. [14] prepared a novel ternary artificial nacre with reinforced ultrathin amorphous alumina that was grown in situ on the surface of graphene oxide (GO) through a vacuum-assisted filtration method. Other methods include freeze casting [15] and electrophoretic deposition [16]. However, all the techniques to produce nacre-like materials have their own shortcomings. Although the biomineralization technology copies the natural process, its drawbacks were that it is so slow that it required a day or more to produce microscale materials [17]. For man-made structural materials manufacturing, such slow growth strategies are not applicable and new time-efficient self-assembly strategies are needed to prepare similarly structured materials [6,17]. The gel-casting method is fast, simple, and versatile; therefore, it can be easily scaled-up and applied to a variety of polymers and platelets combinations to produce composites with excellent mechanical properties [18]. Moreover, the freeze casting method has limitations because it has high demand for the materials [15], and the LBL self-assembly technology and paper-making method require more time and more expensive instruments [19]. Overall, it remains a considerable scientific challenge to find accessible routes to manufacture such large-scale complex materials with high fractions of reinforcements and easily tunable nanoscale structures.

Besides, to prepare one kind of nacre-like material with excellent mechanical properties, the choice of inorganic layered materials congregated with CS is very important [20]. Supported by different technology, Chen et al. [21] inserted different proportions of cationic biopolymer chitosan and quaternate hemicellulose in montmorillonite (MMT) to get compact and robust nanocomposite films with a nacre-like structure and multifunctional characteristics. Liu et al. [22] combined the MMT, nanofibrillated cellulose hydrocolloids, and CS to form hierarchical structure materials with favorable mechanical properties. Zhang et al. [23] prepared CS/GO hydrogel films via water evaporation induced self-assembly method to achieve excellent mechanical properties. Abba [24] integrated CS and Al_2_O_3_ sheets to form thin films with nacre-like brick-and-mortar structure using manual shear casting. The enhanced mechanisms of performance of nacre-like structure materials is related to the complex interaction force between the aforementioned “brick-and-mortar” [25]. Huang’s research group [26] researched the hierarchical structure of nacre-like materials and summarized various enhancement mechanisms to design interfacial interactions between different brick-and-mortar materials. Following this method, they successfully overcame the problems of poor dispersion and weak interfacial interactions in the performance of polymer nanocomposites based on different reinforcement fillers. In addition to the materials mentioned above, other commonly used inorganic layered materials mixed with CS in recently references were including zirconium hydrogenphosphate hydrate [27], hydroxyapatite [28], sepiolite, saponite [29], etc.

It has been reported that the magadiite (MAG) is a kind of sodium polysilicate hydrate that was originally found in the soda lakes in Kenya [30]. At present, magadiite is synthesized in the laboratory through a hydrothermal synthesis method [31]. Compared to the traditionally layered silicates like MMT, magadiite has the following significant advantages. First, magadiite has a more stable layered plate-structure, and its layered plates have a good swelling property, which is suitable for the modifying agents intercalated into it. Second, there are a large number of active functional groups (Si–OH) on the layers of magadiite, and its cation exchange capacity (≈220 mmol/100 g) is greater than MMT (≈80 mmol/100 g) [32]. Third, magadiite has a good compatibility with organic materials, which ensures the functional modification progress. Lastly, different from the parallel layered materials such as the MMT, CaCO_3_, TiO_2_, GO, Al_2_O_3_, Layered Double Hydroxide (LDH), and Al(OH)_3_, the magadiite has the special layered structure like the rose petals-like layered plates [30], which easily formed the “interpenetrating petals” layered composite materials with the polymer, possessing the excellent mechanical and thermal properties [31,33]. Therefore, it is necessary to develop a new kind of inorganic layered materials to prepare the nacre-like materials. In this study, the magadiite with good adsorption and ion-exchange properties was chosen as the “brick” and the CS with great biocompatibility was used as the “mortar” to fabricate a novel nacre-like material. Based on theory above, this research’s novel findings can be highlighted as: (i) the tough and strong nacre-like bio-hybrid CS/MAG membranes with interpenetrating petals structure were fabricated through the gel-casting self-assembling method; (ii) in order to increase the interfacial interactions between MAG nanosheets and CS chains, the γ-aminopropyltriethoxysilane (KH550) was added to the process of fabricating the bio-hybrid CS/MAG membranes for comparison, and based on the contact angle data, the addition of KH550 increased the hydrophobic properties of the membrane; (iii) the mechanical behavior of the nacre-like hybrid membranes was effectively improved, especially tensile strength; (iv) the bio-hybrid membranes had a certain visible light transmittance and possessed an intriguing efficient fire-shielding property during exposure to the flame of an alcohol burner. Besides, it was interesting to further explore some of the other outstanding properties, which have not yet been investigated deeply in other research.

## 2. Experimental

### 2.1. Materials

The magadiite (MAG) was prepared using a hydrothermal synthesis method in the laboratory [31]. The chitosan (CS) was purchased from the Pharmaceutical Group Co., Ltd (Guangzhou, China), and the silane coupling agent γ-aminopropyltriethoxysilane (KH550) was bought from Jinan Xingfei Lung Chemical Co., Ltd (Jinan, China). Other chemical reagents, such as HCl and NaOH, were purchased from Guangzhou Qianhui Company (Guangzhou, China).

### 2.2. Preparation of Nacre-Like Hybrid Membranes with Interpenetrating Petals Structure

#### 2.2.1. Preparation of CS/MAG Nanosheets

A dispersion of MAG in deionized water (1 wt %) was vigorously stirred thoroughly overnight and then centrifuged at 3000 rpm for 10 min to remove unexfoliated MAG. The CS (1 wt %) was dissolved and vigorously stirred for 4 h before use it. The same volume of the exfoliated MAG solution and CS solution (1 wt %) were mixed under the constant stirring for 6 h to guarantee the full adsorption of CS on MAG nanosheets. The CS-coated MAG nanosheets were collected by centrifugation at 3000 rpm for 10 min to remove the supernatant, and finally, the glue-like substance (CS/MAG) was collected.

#### 2.2.2. Preparation of CS/KH550/MAG Nanosheets

A dispersion of MAG in deionized water (1 wt %) was stirred thoroughly overnight and then centrifuged at 3000 rpm for 10 min to remove unexfoliated MAG. Then the KH550 (40 wt % of MAG) was added into the MAG solution dropwise and stirred vigorously for 4 h to fully modify the MAG. Meanwhile, the CS (1 wt %) was also dissolved and vigorously stirred for 4 h before use. Then, the same volume of the KH550/MAG solution and CS solution (1 wt %) were mixed under constant stirring for 6 hours to guarantee the full interaction of CS and KH550/MAG nanosheets. Lastly, the composite nanosheets were collected via centrifugation at 3000 rpm for 10 min to remove the unabsorbed CS and KH550/MAG nanosheets and obtain the glue-like substance (CS/KH550/MAG).

#### 2.2.3. Preparation of the Nacre-Like Hybrid Membranes

The CS/MAG and CS/KH550/MAG nacre-like hybrid membranes with different contents of MAG were fabricated via gel-casting and water-evaporation-induced self-assembly methods. For instance, five CS/MAG nacre-like hybrid membranes with different contents of MAG (the mass ratio of CS vs MAG was 9:1, 8:2, 7:3, 6:4, and 5:5) were prepared using the following process: first, the desired amount of CS/MAG and CS/KH550/MAG glue-like solutions were dispersed under ultrasonication. The obtained suspensions were poured into Petri dishes with a diameter of 9 cm and about 5 mm high, then kept in 50 °C oven for 24 h for evaporation to form the CS/MAG and CS/KH550/MAG nacre-like hybrid membranes with interpenetrating petals structure on the bottom of the Petri dishes (Qianhui Chemical Glass Co., Ltd, Guangzhou, China). In the end, freestanding membranes were obtained by directly peeling off from the bottom of the Petri dishes.

### 2.3. Characterization and Performance of the Nacre-Like Hybrid Membranes

The membranes with different MAG contents were cut into 1 cm × 1 cm squares and immersed in 1 mol/L HCl solution and 1 mol/L NaOH solution for 72 h, and after being dried at 70 °C for 12 h in vacuum oven, the mass change of membranes were measured to analysis the chemical stability. The contact angle of membranes was measured using a fully automatic video micro contact angle measuring instrument (DCa40 MICRO, DATA physics, San Jose, CA, USA). The small angle and wide-angle X-ray diffraction (XRD, D8 ADVANCE, Bruker AXS, Karlsruhe, Germany) studies were performed to assess the presence of crystallites, and the internal structure of the nacre-like membranes was characterized using a scanning electron microscope (SEM, Nova Nano SEM 430, FEI, Hillsboro, OR, USA, where the operating voltage selection was set to 10 kV). The attenuated-total-reflectance-infrared (ATR-IR, NEXUS 670, Thermo Nicolet Corporation, Waltham, MA USA) spectroscopy was used to evaluate the chemical structure of the membranes. The thermal stability of the membranes was examined using thermal gravimetric analysis (TGA, STA449 C, NETZSCH, Selb, Germany) and differential thermal gravimetric (DTG). Apart from the mechanical properties (Instron 5566, Instron, Boston, MA, USA), the properties of transparency (UV-2000, Jiapeng Technology Co., Ltd. Shanghai, China) and fire resistance had been investigated.

## 3. Results and Discussion

### 3.1. Chemical Stability and Contact Angle Analysis

**Figure 1:**
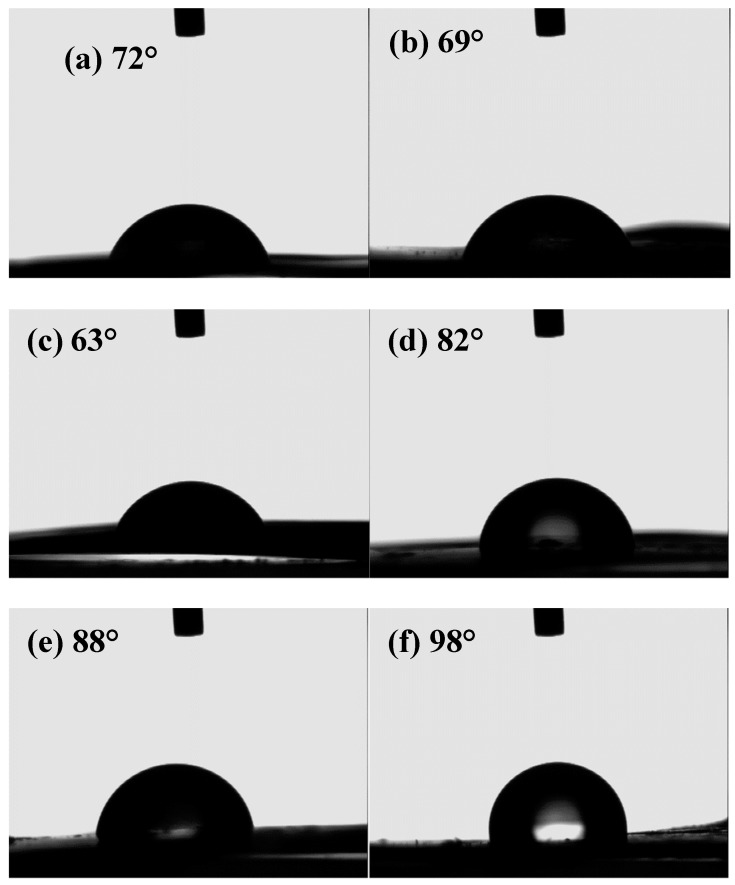
Pictures of water droplets on chitosan/magadiite (CS/MAG) (**a**–**c**) and CS/KH550/MAG (**d**–**f**) membranes with different mass proportion for contact angle. (a,d), (b,e), (c,f) for CS:MAG = 9:1; 7:3; 5:5, respectively.

Figure 1 shows the pictures of water droplets on CS/MAG (a–c) and CS/KH550/MAG (d–f) membranes with different weight proportions for the contact angle. It is seen that the CS/MAG membranes were more hydrophilic than the CS/KH550/MAG membranes, and with the increasing content of MAG in CS/MAG, the contact angle kept go down. This indicates that MAG is more hydrophilic than CS and the incorporation of KH550 into CS/MAG made the membrane hydrophobic [34]. This was because, first, MAG is a mesoporous clay material whose petals have a strong binding force with water molecules [32]. Second, the special molecular structure of KH550 played an important role to combine the CS chain with a brick of MAG via the formation of a covalent bond [26] and van der Waals forces. It is also interesting to notice that in the case of the CS/KH550/MAG composite membranes, the contact angle increased with MAG content, meaning it became more hydrophobic, which also could be explained by the KH550 molecular structure.

The chemical stability property was characterized using the weight change, and the mass loss ratio (L) was calculated using the following equation:

(1) $$L\left(\%\right)=\frac{M_{0}-M_{t}}{M_{0}}\;\times 100\%$$

As shown in Table 1, it was found that the nacre-like composite membranes lost more weight in acid conditions than alkaline, which indicated the membranes had stronger chemical stability in an alkaline environment. Compared with CS/MAG membranes, it is interesting to find that the weight loss ratio of CS/KH550/MAG membranes decreased with the increasing content of MAG whatever the conditions, and the CS/MAG lost similar mass with the CS/KH550/MAG membranes in acid conditions, but it was decreased in an alkaline environment. It can be concluded that the CS and KH550 were so unstable in an acidic environment that the nacre-like structure of membranes were out of action. Considering the chemical nature of the individual components, first, the CS molecular was soluble in acid, which resulted in a portion of weight change [35]. On the other hand, the functional group –NH_2_ in the molecular structure of KH550 reacted with the acid solution. According to the previous reference [24], the MAG inorganic layers had excellent stability whose inorganic layers could hinder the diffusion action of solute molecules when it formed the "interpenetrating petals" layered structure with CS and KH550. Overall, the CS/KH550/MAG nacre-like structure membranes held good chemical stability in alkaline conditions.

### 3.2. XRD Analysis

**Figure 2:**
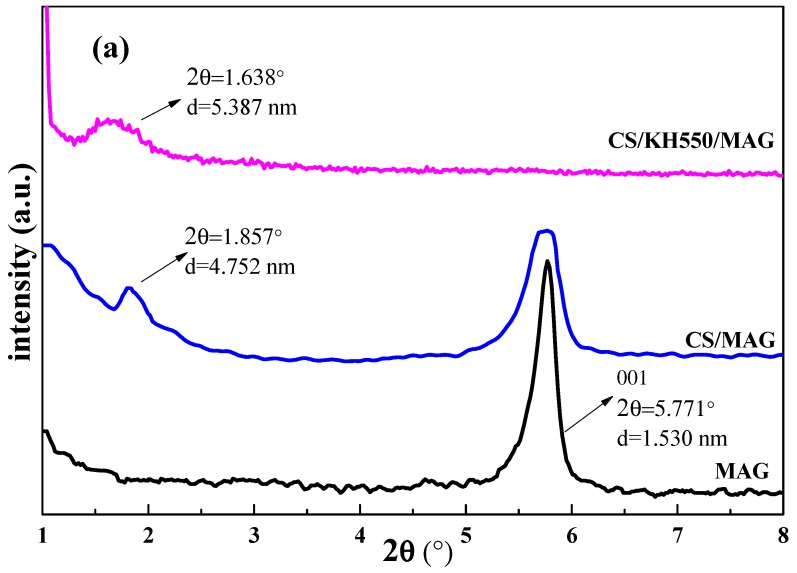

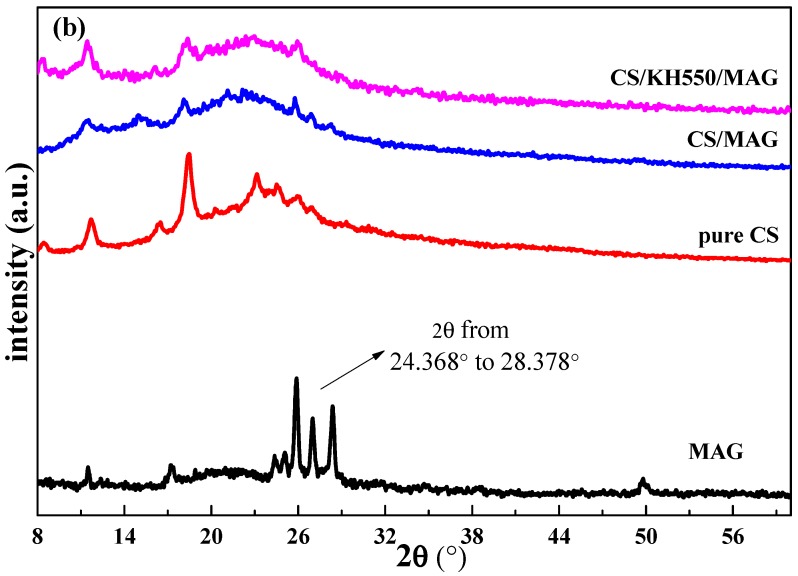
The (**a**) small angle and (**b**) wide-angle X-ray diffraction (XRD) patterns of CS/MAG and CS/KH550/MAG hybrid membranes.

Figure 2 shows that the small-angle and wide-angle XRD spectrums of the CS/MAG and CS/KH550/MAG hybrid membranes were tested, respectively. The Figure 2a shows that in the small-angle XRD spectrums, the 001 diffraction peaks of the CS/MAG and CS/KH550/MAG hybrid membranes moved in the direction of the small angle compared to the pure MAG [32], which demonstrated that both the hybrid membranes possessed the layered structure and their layered spacing was larger than the pure CS membrane and their layered spacings were 4.752 nm, 5.387 nm, and 1.530 nm. Because a large number of CS molecular chains were adsorbed into the layered structure, the organic and inorganic assembled in an orderly manner in the hybrid membranes. However, the CS/MAG membrane still had an obvious diffraction peak at 2θ = 5.771°, while the CS/KH550/MAG membrane did not perform similarly. It was shown that there were still some parts of the MAG layers not being inserted by the CS molecular chains, but the intercalated reaction of CS/KH550/MAG hybrid membranes was more complete after adding the KH550.

The diffraction peaks from pure CS at 2θ = 8.445 °, 11.757 °, and 18.362° were shown in both kinds of hybrid membranes. However, the typical diffraction peaks of MAG in both the hybrid membranes at 2θ = 24.368°–28.378° almost disappeared. It was shown that the CS molecular chains were inserted into the MAG layers in both kinds of hybridized membranes. The distance between the layers became larger, and the crystal structure of MAG was significantly damaged. As we could see, the spacing between the layers was much larger and the insertion reaction was more complete after adding the KH550. Because the silicon alcohol from KH550 after hydrolysis would easily react with the functional groups of Si–OH on the MAG, which improved the lipophilicity of the MAG layers and the dispersion of MAG in the CS matrix, and enhanced the cohesive force between the MAG and CS, thus making a better connection [36].

### 3.3. SEM Analysis

**Figure 3:**
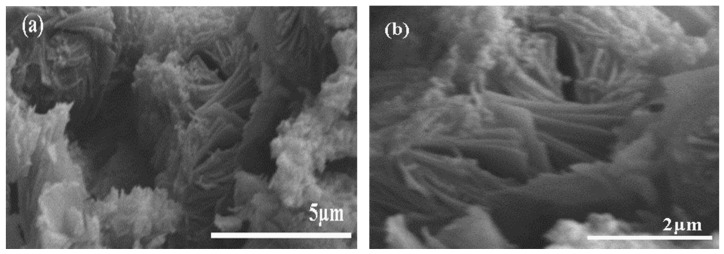

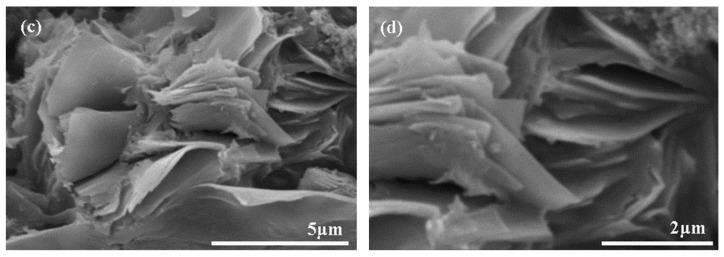
Cross section scanning electron microscope (SEM) images of CS/MAG (**a**,**b**) and CS/KH550/MAG hybrid membranes (**c**,**d**).

Figure 3 shows the cross-sectional SEM pictures of CS/MAG and CS/KH550/MAG hybrid membranes. Consistent with the experimental results of the XRD analysis, both hybrid membranes indeed possessed the distinct layered structure. It was determined from the cross-section SEM pictures that both hybrid membranes were fabricated by the densely layered structures from the MAG layers and the CS matrix. However, there was a difference between the so-called “interpenetrating petals” structure and the “bricks and mortar” structure in imitating the nacre materials [3]. Researchers have reported that unlike inorganic layered materials, such as MMT, CaCO_3_, TiO_2_, GO, Al_2_O_3_, LDH, and Al(OH)_3_, the layers of MAG were like the rose petals [30].

The other inorganic materials of the “bricks and mortar” structure were just flat, while the MAG from the “interpenetrating petals” structure had a certain bending deflection. This was because of the interpenetrating and hybridization between the MAG layers and CS molecular chains, which led to the formation of a regular and compact layered structure inside the nacre-like hybrid membranes [37]. The “interpenetrating petals” layered structure inside of the nacre-like hybrid membranes were constituted by the solid MAG layers and flexible CS molecular chains completely; when the whole hybrid membrane suffered from the tensile forces, the pulling difficulty of the MAG layers would increase because of the blocking effect from the “petal-like” layers [4,27].

The cross-section SEM pictures of the CS/MAG hybrid membranes are shown in Figure 3a,b, while Figure 3c,d represent the cross-sectional SEM pictures of the CS/KH550/MAG hybrid membranes. Like the result of the XRD analysis, the MAG layers in the CS/KH550/MAG hybrid membranes were inserted more completely and deeply than those in the CS/MAG hybrid membranes, and the layered spacing of the CS/KH550/MAG hybrid membranes was much larger compared to the CS/MAG hybrid membranes. From the angle of the microstructure, the interfacial compatibility between the MAG layer and the CS molecular chain was significantly enhanced after adding the KH550 and which was made the CS reaction to insert in the molecular chains [25].

Having a close-up view of the cross-section SEM images of CS/MAG and CS/KH550/MAG hybrid membranes in Figure 3, it was found that the MAG layers in the CS/KH550/MAG hybrid membranes had a more visible status of pulling out, and the deflection and curvature of the MAG layers were more obvious than the CS/MAG hybrid membranes. On the one hand, the reasons were found to be the rose petal-like MAG layers indeed suffered greatly from the resistance of being pulled out as well as the strong cohesive forces between MAG layers and CS molecular chains led the hybrid membranes more condensed. By adding the silane coupling agent KH550, which improved the interfacial properties and the resistance of MAG layers, the mechanical properties of CS/KH550/MAG hybrid membranes would be significantly improved [37].

### 3.4. Fourier Transforms Infrared Spectroscopy (FTIR) Analysis

**Figure 4:**
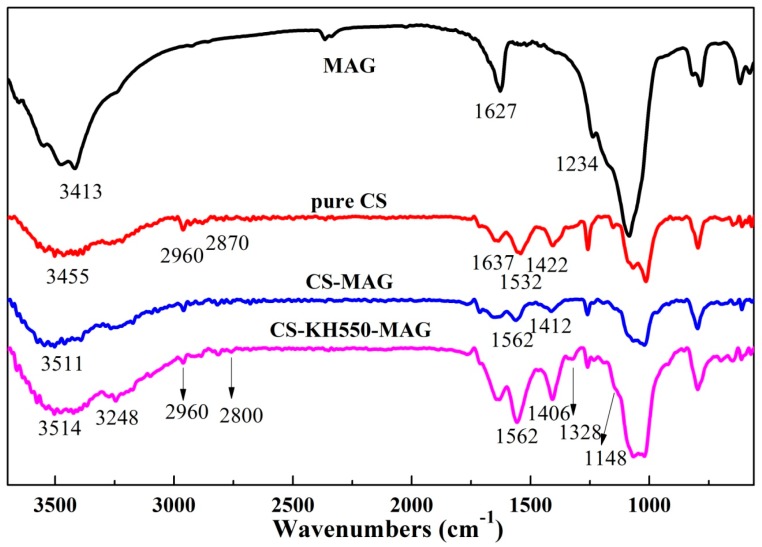
Fourier transforms infrared spectroscopy (FTIR) spectra of CS/MAG and CS/KH550/MAG hybrid membranes.

The high and low frequency of the absorption peak in the infrared spectrum showed the change of atomic intensity in the MAG lattice, while the shift of the absorption peak had been reflected due to the distortion of the intrinsic MAG lattice. It had been shown that there were some kinds of molecules moving into the MAG layers and changing the distribution of the structural force inside the MAG. Figure 4 shows the infrared spectra of both of the CS/MAG and CS/KH550/MAG hybrid membranes.

As shown in Figure 4, compared to the pure CS membranes, the stretching vibration peak of N–H bonds located at 3455 cm^−1^ belonged to the CS blue shifted to 3511 cm^−1^ in the CS/MAG hybrid membranes and the width and intensity of this absorption peak was increased. Researchers have reported that the –NH_2_ functional groups in the CS could easily absorb H^+^ to form –NH_3_^+^ because it was mainly influenced by the Si–OH functional groups on the MAG layers, which reacted with the negatively charged Si–OH functional groups [34]. Besides, the H of the –NH– in the CS and the O of the Si–OH functional groups could easily form the hydrogen-bond interaction [38]. It was the electrostatic effect between the negative MAG layers and the protonated –NH_3_^+^ on the CS that resulting in the blue shifting of the deformation vibration absorption peak of –NH_3_^+^ on the CS from 1532 cm^−1^ to 1562 cm^−1^. The change of the absorption peak above illustrated that there was a strong force between the CS molecular chains and MAG inorganic layers in the CS/MAG hybrid membranes, which could combine the CS and MAG together very well.

As for CS/KH550/MAG hybrid membranes, except the above changes showed like CS/MAG hybrid membranes, there were some other changes in the absorption peaks. The increased intensity of the absorption peak at 3248 cm^−1^ indicated that the silane coupling agent KH550 reacted with the Si–OH functional groups on the MAG layers, which strengthened the lipophilicity of the MAG layers and enhanced the compatibility of CS organic molecular chains and MAG inorganic layers [39]. The weakened spectra are shown at the range of 2800~2960 cm^−1^, which represented the –CH retractable peak of the –CH_2_ in the silane coupling agent KH550. The increased intensity of the absorption peak at 1562 cm^−1^ illustrated that the chemical bonds and electrostatic interactions between the negative MAG layers and the protonation –NH_3_^+^ functional groups on the CS molecular chains after adding the KH550. Because of that, on the one hand, the silanol groups from the hydrolysis of KH550 would form the coupling effect, simultaneously reacted with the Si–OH functional groups on the MAG and the –NH_3_^+^ functional group’s reaction on the CS molecular chains; on the other hand, the –NH_2_ functional groups on the KH550 could easily form the hydrogen bonds with the Si–OH on the MAG layers or the –OH on the CS molecular chains. The increased intensity at 1406 cm^−1^ indicated that the deformation vibration of –C–H bonds of CH_3_ and CH_2_ were more obvious owing to the static electricity or hydrogen bonding interactions. The new emerging characteristic peak at 1328 cm^−1^ was belonged to –Si–O–C– group, which showed the formation of chemical bonds between the KH550 and CS molecular chains [40]. Furthermore, the weak shoulder peak at 1148 cm^−1^ belonged to the opponent contraction vibration and the bending vibration peak of Si–O–Si formed from the Si–OH on the KH550 and MAG. Therefore, through these above changes, adding the silane coupling agent KH550 not only improved the interface state and the compatibility of MAG layers and CS molecular chains but also could form a stronger coupling reaction with both the MAG layers and CS molecular chains [37].

**Figure 5:**
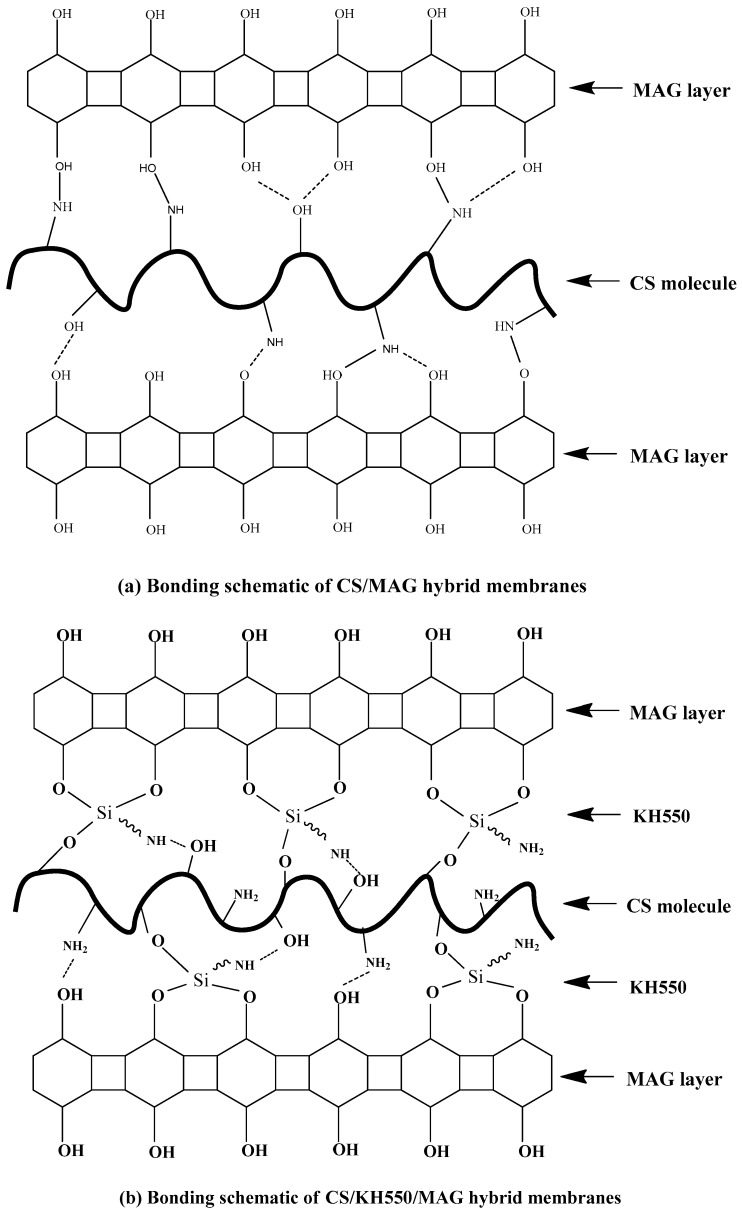
The bonding schematics of (**a**) CS/MAG and (**b**) CS/KH550/MAG hybrid membranes.

Figure 5 clearly shows the bonding schematics of both the CS/MAG and CS/KH550/MAG hybrid membranes. The CS/MAG hybrid membranes were combined via chemical bonds and hydrogen bonds, and so were the CS/KH550/MAG hybrid membranes. The coupling reaction of KH550 between MAG and CS made more complicated bonds and a stronger bonding force.

### 3.5. Mechanical Properties Analysis

**Figure 6:**
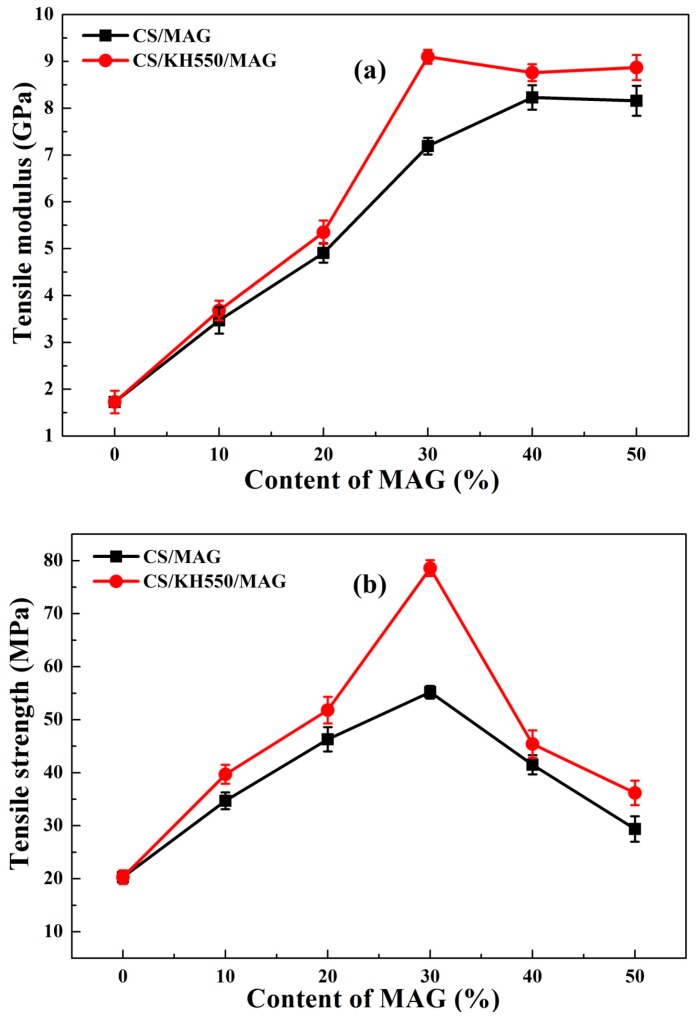

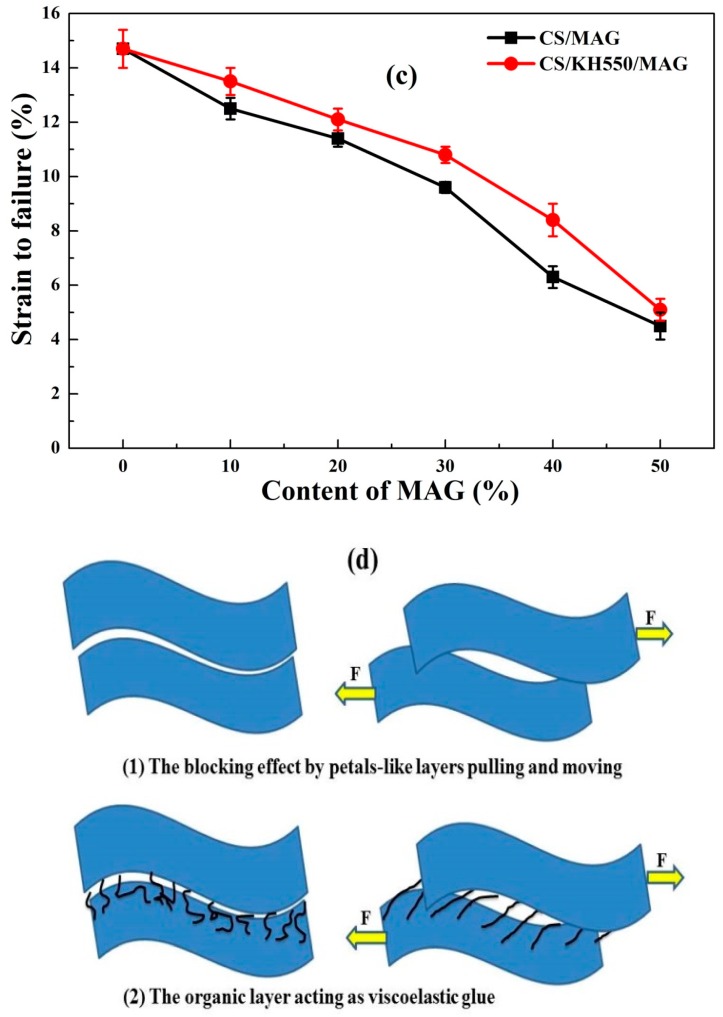
Mechanical properties of (**a**) tensile modulus, (**b**) tensile strength, (**c**) strain to failure, and (**d**) force schematic diagram of MAG layers pulling and moving of the CS/MAG and CS/KH550/MAG hybrid membranes.

Figure 6 shows the mechanical properties of hybrid membranes with different content of MAG. As shown in Figure 6, when the quality ratio of CS and MAG was equal to 7:3, the tensile strength of both the CS/MAG and CS/KH550/MAG hybrid membranes reached the maximum. Under this proportion of quality ratio of CS and MAG, the tensile modulus and tensile strength of the CS/MAG hybrid membranes reached 7.19 GPa and 55.2 MPa, respectively, which were 4.16 and 2.72 times higher than the pure CS membranes (when the content of MAG is 0), respectively. The tensile modulus and tensile strength of the CS/KH550/MAG hybrid membranes reached 9.10 GPa and 78.6 MPa, respectively, which were 5.26 and 3.87 times of the pure CS membranes, respectively. The tensile strength of the CS/KH550/MAG hybrid membranes when the quality ratio of CS and MAG was equal to 7:3 in this study had been achieved in the range of nacre-like materials (70–130MPa) reported in other works of literature [3,4]. However, the strain to failure of the CS/MAG and CS/KH550/MAG hybrid membranes could still remain 9.6% and 10.8% at the quality ratio of CS and MAG to 7:3.

The above data of mechanical properties has shown that the “interpenetrating petals” layered structure, based on the MAG layers, could significantly improve the mechanical properties of the pure CS membranes. The hybrid membranes not only had possessed an excellent tensile strength but also could maintain the great toughness and ductility, which was confirmed that the nacre-like materials have ultra-strong and highly condensed characteristics [26]. Comparing to other data of the mechanical properties with the same quality content of MAG, it was shown that, after adding the silane coupling agent KH550, both the tensile modulus and tensile strength of CS/KH550/MAG hybrid membranes were promoted compared to the CS/MAG hybrid membranes, which illustrated that the KH550 could enhance the intermolecular forces between the MAG layers and CS molecular chains [34].

In order to explain why the “interpenetrating petals” layered structure, based on the MAG, had an excellent effect on the mechanical properties of the nacre-like hybrid membranes, Figure 6d shows the force schematic diagram of MAG layers pulling and moving that can be understood from the following three points. First, there was a blocking effect from the petal-like layers pulling and moving. When the hybrid membranes suffered tensile stress, the petal-like interspersed layers of MAG cause the joint interlock effect of mortise and tenon in the process of slipping out [33]. However, the MAG layers with a certain deflection stuck to each other, so the stable MAG inorganic layers could change the direction of force transmission, which made the stress more even inside the hybrid membranes, which then enhanced the tensile strength [41]. Second, there was an effect of cementing action from the organic substrate. The organic matrix like the CS bonded closely together with MAG layers through the chemical bonds, hydrogen bonds, or electrostatic forces [25]. When the hybrid membranes were suffering the plastic deformation, on the one hand, a large number of chemical bonds and hydrogen bonds would be destroyed and restructured during the MAG layers sliding out, and the effect of cementing action from the organic substrate would improve the slip resistance, effectively maintaining the “interpenetrating petals” layered structure inside the hybrid membranes, and delaying the fracture time [26]. On the other hand, the effect of cementing action from the organic matrix could also prevent the propagation of the crack, so as to reach an excellent toughness. Third, the silane coupling agent KH550 worked as the bridge role in the CS/KH550/MAG hybrid membranes. The KH550 not only could improve the interfacial properties and compatibility between MAG and CS, but also could raise the coupling reaction to form the stronger force. As a bridge between MAG layers and the CS organic matrix, the KH550 further improved the mechanical properties of hybrid membranes. Therefore, the reason that the “interpenetrating petals” layered structure had an excellent effect on the mechanical properties of the nacre-like hybrid membranes was the comprehensive results of the above mechanism.

### 3.6. Thermal Properties Analysis

**Figure 7:**
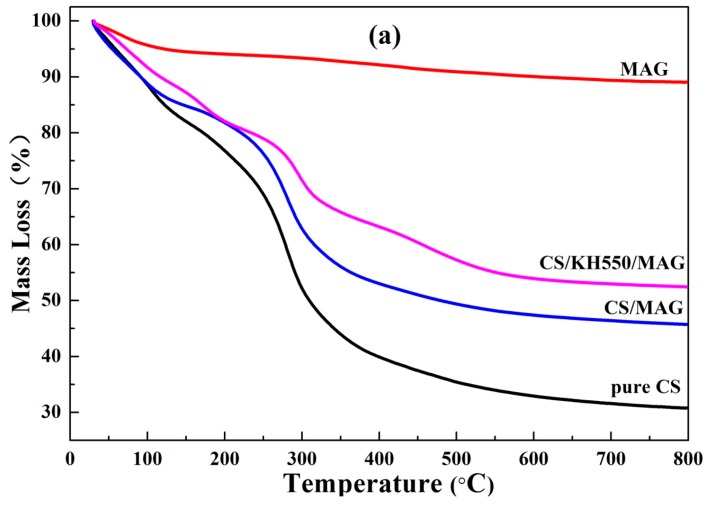

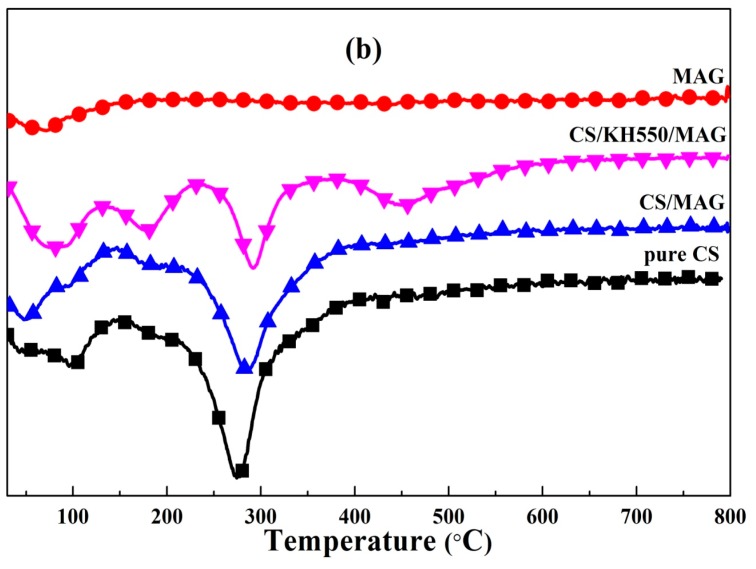
(**a**) Thermal gravimetric (DTG) and (**b**) differential thermal gravimetric (DTG) curves of MAG, pure CS, CS/MAG, and CS/KH550/MAG hybrid membranes.

Figure 7 shows the TG and DTG curves of MAG, pure CS membranes, CS/MAG, and CS/KH550/MAG hybrid membranes. From Figure 7, two stages in the decomposition of pure MAG can be seen. The apparent adsorption water and bound water were stripped from 30 to 250 °C; the condensation reaction of Si–OH in the MAG was condensed into the siloxane mainly from 250 to 800 °C. The weightlessness of the pure CS membrane, CS/MAG, and CS/KH550/MAG hybrid membranes were divided into three stages. The apparent adsorption water and bound water were mainly stripped from 30 to 200 °C, and the weightlessness of thermal decomposition of the CS molecular chains and KH550 was mainly from 200 to 500 °C. At temperatures from 500 to 800 °C, the weightlessness of pure CS membrane was the stripping of pyrolysis products, but the weightlessness of CS/MAG and CS/KH550/MAG hybrid membranes not only included the removal of the thermal decomposition of organic matrix but also contained the condensation reaction of silicon hydroxyl in the remaining MAG layers.

At temperatures from 200 to 500 °C, the maximum weightlessness temperature of the pure CS, CS/MAG, and CS/KH550/MAG hybrid membranes was 274.3 °C, 285.5 °C, and 296.8 °C, respectively. It was illustrated that the “interpenetrating petals” layered structure, based on the MAG layers, heightened the thermal stability of CS membranes, and the silane coupling agent KH550 created the higher thermal stability of the CS/KH550/MAG hybrid membranes [26]. As shown in Figure 7, there was a weightlessness peak near 450 °C in the DTG curve of the CS/KH550/MAG hybrid membranes, which indicated that the CS/KH550/MAG hybrid membrane possessed a higher thermal stability than that of the CS/MAG hybrid membrane.

The reason that the “interpenetrating petals” layered structure could heighten the thermal stability of CS membranes can be understood by the following three points [25,34] as (i) in the hybrid membranes with the “interpenetrating petals” layered structure, the CS molecular chains were intercalated into the interlayers of MAG, so the MAG layers possessed excellent thermal stability and a barrier property, such that it could raise the thermal degradation temperature of CS molecular chains to a higher degree; (ii) the hydrogen bonds and electrostatic force between the MAG layers and CS molecular chains could enhance the thermal stability of the hybrid membranes; and (iii) in the CS/KH550/MAG hybrid membranes, the addition of KH550 could not only make the intercalated reaction more complete but could also raise the coupling reaction to form the stronger force, so as to further improve the thermal stability of the hybrid membranes.

### 3.7. Transmission Analysis

**Figure 8:**
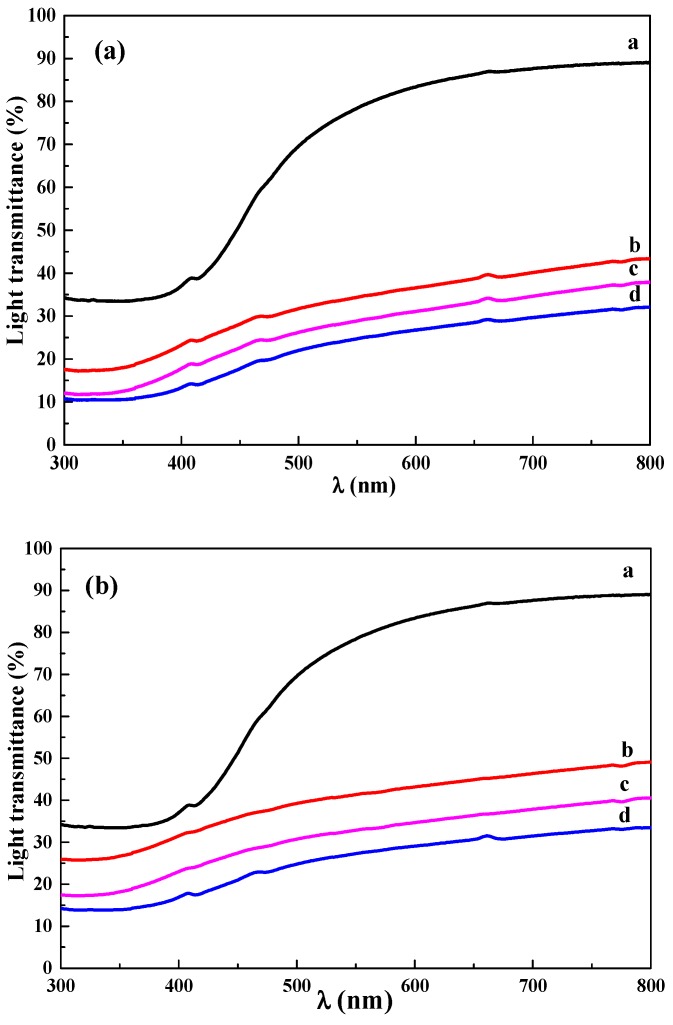
The UV-vis transmission spectra of CS/MAG (**a**) and CS/KH550/MAG (**b**) hybrid membranes with different quality proportion, a for pure CS membrane, and b, c, and d for CS: MAG = 9:1; 7:3; 5:5, respectively.

In order to investigate the light transmission of the hybrid membranes, an ultraviolet spectrophotometer was used to test the pure CS membrane and both hybrid membranes with different proportions of MAG. As shown in Figure 8, the transmittance of visible light area (400–760 nm) of the pure CS membrane was from 37% to 88%, and that of the CS/MAG and CS/KH550/MAG hybrid membranes was from 10% to 42% and from 15% to 50%, respectively. It was illustrated that even if adding the non-transparent MAG layers, the hybrid membranes still possessed a certain transmission of light. Comparing the curves of Figure 8a,b, the visible light transmittance of CS/KH550/MAG hybrid membranes under the same quality ratio of MAG was a little higher than the CS/MAG hybrid membranes, which illustrated that adding KH550 in the hybrid membranes increased the orientation of the “interpenetrating petals” layered structure [42].

**Figure 9:**
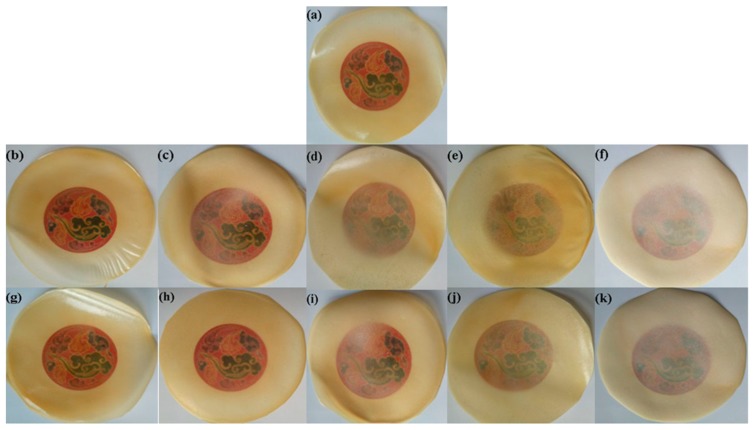
The optical pictures of pure CS membrane (**a**), CS/MAG (**b**–**f**) and CS/KH550/MAG (**g**–**k**) hybrid membranes with different quality proportion, with (b–f) and (g–k) both corresponding to CS: MAG = 9:1; 8:2; 7:3; 6:4; 5:5, respectively.

The optical photographs of the pure CS membrane, CS/MAG, and CS/KH550/MAG hybrid membranes are shown in Figure 9. These membranes were flexible, glossy, and their surfaces were very smooth. It was shown that the visible light transmittance of the hybrid membranes fell with the increasing content of MAG layers, but that of the CS/KH550/MAG hybrid membranes was a little better than CS/MAG hybrid membranes with the same MAG proportion.

### 3.8. Burning Experimental Analysis

**Figure 10:**
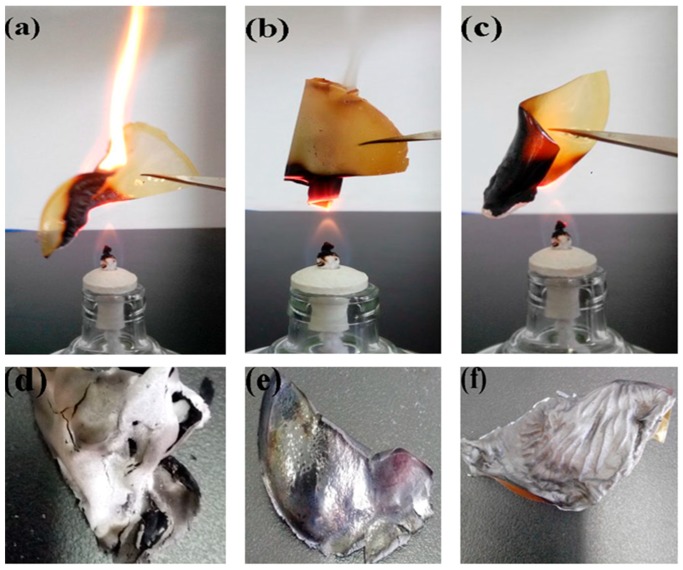
The burning and burned pictures of pure CS (**a**,**d**), CS/MAG (**b**,**e**), CS/KH550/MAG (**c**,**f**) hybrid membranes.

In terms of the functional benefits of these nacre-like membranes with an interpenetrating petal structure, preliminary experiments on their fire-retardant property was also conducted. The photographs depicting burning and burned pure CS and both hybrid membranes were shown in Figure 10, which demonstrated the intriguing efficient fire-shielding properties of the hybrid membranes during exposure to the flame from an alcohol burner.

As shown in Figure 10, when exposed to the flame, the pure CS membrane instantly curled up and quickly burned fiercely with a large combustion flame, but the surface of the CS/MAG and CS/KH550/MAG hybrid membranes gradually darkened during burning, mainly induced by carbonization of the CS molecules. With the extended time in the flame, the pure CS membrane burned quickly with a snapping noise and smoke, but there did not appear to be any burning flame when the CS/MAG and CS/KH550/MAG hybrid membranes were exposed to the flame. The bending degree of the CS/MAG hybrid membrane was more obvious than that of the CS/KH550/MAG hybrid membrane. In addition, the CS/KH550/MAG hybrid membrane had no dense smoke when burning on the alcohol burner. Furthermore, as expected, the whole procedure of burning both the CS/MAG and CS/KH550/MAG hybrid membranes never led to any dripping of hot fluids like the other plastic membranes, and the shape of the CS/KH550/MAG hybrid membrane was maintained even with constant exposure to the flame of the alcohol burner.

It can be seen from the above combustion experiment phenomenon that the MAG hybrid membranes with the "interpenetrating petals" layered structure had a certain fire-retardant performance, and the addition of KH550 could improve the fire-retardant performance of the hybrid membranes [43]. This can be understood from the following three reasons. First, the MAG inorganic layers in the "interpenetrating petals" layered structure could form lots of dense fire-retardant layers, which could hinder the flame and gas diffuser [43]. Therefore, it could protect the CS molecular chains in the hybrid membranes because of the lack of oxygen. Second, when exposed to the flame of the alcohol burner, the dihydroxylation of Si–OH on the MAG layers would produce water, and the evaporation from hybrid membranes would take away a lot of heat. Therefore, the heat losses would make it difficult to reach the ignition point of the CS and could not support the combustion of the hybrid membranes. Third, adding the silane coupling agent KH550 made the CS molecular chains intercalate more fully and deeply in the CS/KH550/MAG hybrid membrane, which possessed a denser layered structure than the CS/MAG hybrid membrane and further enhanced the fire-retardancy of the hybrid membranes [40].

Compared to the residues of combustion, the pure CS membrane became brittle. Bubbles appeared on the surface of the CS/MAG hybrid membrane, which were caused by the removal of moisture during the process of combustion. The surface of the CS/KH550/MAG hybrid membrane appeared as clear layered stripes because of the neater layers in the CS/KH550/MAG hybrid membrane and the coupling force of the interpenetrating layered structure made the self-supporting ability stronger.

Therefore, the “interpenetrating petals” layered structure in the hybrid membranes had a certain stability and fire resistance [44]. Additionally, the hybrid membranes did not contain heavy metals and halogen atoms, which are often needed in present-day flame-retardant materials [45]. Taking into account that the preparation could be easily scaled to large quantities, we expect that this effort contributes toward producing environmentally friendly, viable alternatives for future flame-retardant material.

## 4. Conclusions

It can be concluded that nacre-like hybrid membranes with unique interpenetrating petals structure were fabricated. These organic and inorganic composites were tough and strong with certain fire-shielding and excellent mechanical properties. The MAG layers were deeply inserted to increase the layer spacing by the CS molecular chains, which formed the dense layered interpenetrating structure. The static electricity and hydrogen bonding interactions networks were established among the hybrid membranes, and the silane coupling agent KH550 worked as the bridge role to improve the interfacial properties and to form the stronger force through the coupling reaction. Therefore, our designed multiscale assembly is an alternative approach to prepare strong functional hybrid materials, and the nacre-like hybrid membranes are very promising in the construction of highly tough and strong composites to be applied in biomaterial research, such as tissue engineering in biomedical research.

## Figures and Tables

**Table 1:** The mass change of CS/MAG and CS/KH550/MAG after 72 h in acidic or basic environments (the concentration of HCl and NaOH solutions were 1 M, and the squares of membranes were 1 cm × 1 cm).

Content of MAG		CS/MAG			CS/KH550/MAG		
		10%	30%	50%	10%	30%	50%
Acid	Beginning (g)	0.0180	0.0177	0.0138	0.0182	0.0161	0.0174
	After 72 h (g)	0.0134	0.0136	0.0114	0.0140	0.0135	0.0139
	L (%)	25.56%	23.16%	17.39%	23.08%	16.15%	20.11%
Basic	Beginning (g)	0.0183	0.0137	0.0135	0.0175	0.0174	0.0194
	After 72 h (g)	0.0149	0.0125	0.0115	0.0143	0.0159	0.0185
	L (%)	18.58%	8.76%	14.8%	18.28%	8.62%	4.64%
